# FOXM1 Targets TRIM59 to Activate Wnt/β-Catenin in Tumor Stemness and Hepatoblastoma Metastasis

**DOI:** 10.3390/ijms27219843

**Published:** 2026-11-03

**Authors:** Hanlei Yan, Wei Liu, Wanying Jia, Jingjing Huang, Wenyao Xu, Zihao Fu, Baijun Zheng, Hui Yu, Xinlin Chen, Ya Gao, Donghao Tian

**Affiliations:** 1 Department of Pediatric Surgery, The Second Affiliated Hospital of Xi’an Jiaotong University, No 157, Xi Wu Road, Xi’an 710004, China; yanhl1002@163.com (H.Y.);; 2 Institute of Neurobiology, Environment and Genes Related to Diseases Key Laboratory, Xi’an Jiaotong University, No 96, Yan Ta Xi Road, Xi’an 710061, China

**Keywords:** hepatoblastoma metastasis, tumor stemness, Wnt/β-catenin signaling, epithelial-mesenchymal transition

## Abstract

Hepatoblastoma is the most common liver malignancy in children. Approximately 20% of patients present metastasis at diagnosis and have a dismal prognosis. However, the molecular mechanisms and targeted therapeutic strategies remain limited. Integrated analysis of GSE151347, GSE133039, GSE131329 and GSE75271 datasets, incorporating the mRNA expression-based stemness index, weighted gene co-expression network analysis, ChEA3 and TRRUST databases, receiver operating characteristic (ROC) curve and differential expression analysis, identified forkhead box protein M1 (FOXM1) as a key transcription factor significantly correlated with metastatic hepatoblastoma and poor clinical outcomes. In HuH6 and HepG2 cells, FOXM1 promoted stem cell-like phenotype, migration, invasion and epithelial–mesenchymal transition. In vivo, FOXM1 promoted tumor growth in a subcutaneous xenograft model and lung colonization in a tail-vein injection model. The CUT&Tag sequencing revealed that FOXM1 occupied the tripartite motif containing 59 (*TRIM59*) promoter, and dual-luciferase reporter assays confirmed that FOXM1 transcriptionally activated *TRIM59*. Furthermore, FOXM1 gain- and loss-of-function were accompanied by changes in axis inhibition protein 1 (AXIN1) protein levels, β-catenin levels, and β-catenin nuclear translocation, potentially contributing to the regulation of Wnt/β-catenin signaling. Finally, TRIM59 knockdown successfully rescued FOXM1-driven malignant phenotypes. This study identifies a novel FOXM1/TRIM59/Wnt signaling axis that drives hepatoblastoma metastasis, highlighting FOXM1 and TRIM59 as promising therapeutic targets for high-risk patients.

## 1. Introduction

Hepatoblastoma (HB) is the most common pediatric liver malignancy, with a rising incidence particularly among children aged 2–4 years [1,2]. Although intensive chemotherapy combined with surgical resection has improved long-term survival [3], approximately 20% of patients present metastasis at diagnosis, and remain a dismal prognosis [4]. Moreover, systemic therapy-related toxicity has been associated with impaired quality of life in patients with hepatoblastoma, underscoring the urgent need for deeper mechanistic understanding and targeted therapeutic strategies [5,6].

Single-cell transcriptomic analyses have supported a cancer stem cell (CSC) model for hepatoblastoma [7], which is widely considered to be the primary driver of tumor metastasis and recurrence [8,9]. Consistently, metastatic hepatoblastoma frequently exhibits an enriched tumor stemness signature [10], in which the canonical Wnt/β-catenin pathway is recognized as the core regulator [11,12]. Hepatoblastoma is characterized by a remarkably poor tumor mutational burden (TMB), although emerging evidence indicates that non-genetic factors, including histone acetylation modification, post-translational protein stability and the ubiquitin–proteasome system contribute to hepatoblastoma progression, although this is lacking effective clinical translation [12,13]. The precise molecular mechanisms by which the Wnt signaling pathway maintains stemness are not fully understood, and no targeted interventions are currently available. Forkhead box protein M1 (FOXM1) is a well-established transcription factor that orchestrates multiple malignant hallmarks, including tumor stemness and metastasis [14]. The E3 ubiquitin ligase tripartite motif containing 59 (TRIM59) has emerged as a critical regulator of protein degradation pathways in several cancers [15]. Despite these insights, the mechanisms by which FOXM1 and TRIM59 contribute to hepatoblastoma progression, particularly their relationship with Wnt signaling, remain incompletely understood.

In this study, we utilized integrated bioinformatic screening followed by experimental validation to identify a novel FOXM1/TRIM59/Wnt signaling cascade contributing to hepatoblastoma metastasis. Our findings suggest that FOXM1 transcriptionally upregulates *TRIM59* and modulates Wnt/β-catenin signaling activity, possibly through affecting AXIN1 protein levels. By this signaling cascade, our data raise the possibility that Wnt signaling may be further amplified in the context of CTNNB1 mutations (the gene encoding β-catenin). This study identifies a highlighted signaling axis contributing to hepatoblastoma progression, and presents a promising therapeutic target for metastatic patients.

## 2. Results

### 2.1. Identification of Hub Stemness-Related Genes and Transcriptional Regulatory Networks Analysis in Hepatoblastoma

**Figure 1:**
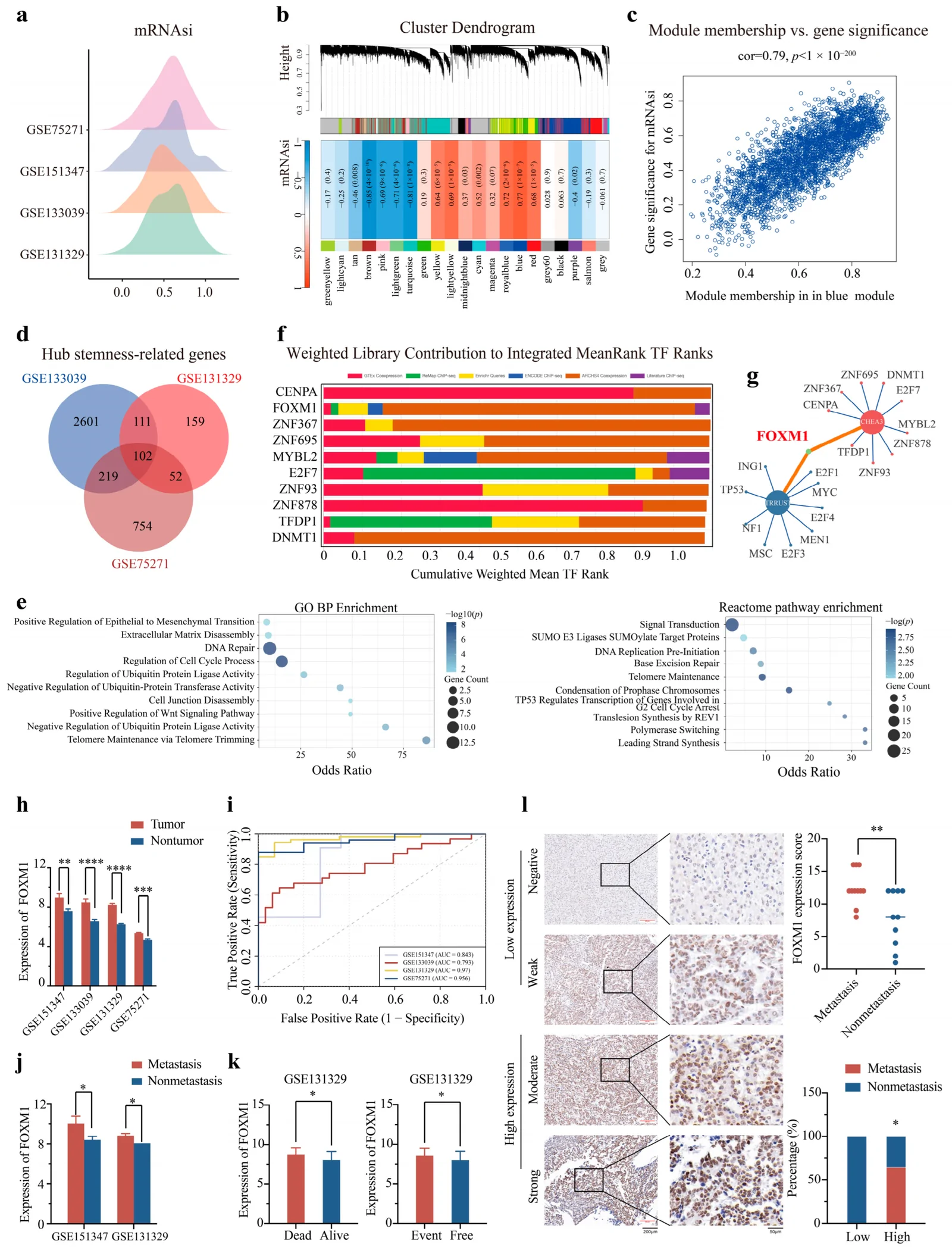
Identification of hub stemness-related genes and the clinical correlation analysis of FOXM1 expression in hepatoblastoma. (**a**) The mRNA expression-based stemness index (mRNAsi) of 4 datasets, where the higher values correspond to more pronounced stemness. (**b**) The WGCNA analysis showed gene modules being correlated with mRNAsi. (**c**) Scatter plots illustrate the relationship between the Module Membership and Gene Significance. (**d**) A Venn diagram of the 102 common stemness-related hub genes, by intersecting the stemness-associated modules from 3 datasets. Functional enrichment analysis of 102 hub genes by GO Biological Process and Reactome pathway (**e**). (**f**) The candidate TFs for the 102 hub genes were screened via the ChEA3 platform. (**g**) Transcriptional regulatory networks analysis showed the FOXM1 as the only transcription factor that was consistent in both CHEA3 and TRRUST database. (**h**) FOXM1 expression in hepatoblastoma tissues compared to non-tumor liver tissues. (**i**) The ROC curve showed AUC values from 0.793 to 0.970. (**j**,**k**) FOXM1 expression according to metastasis status (non-metastatic vs. metastatic), patient survival (dead vs. alive), and event-free survival (with vs. without events). (**l**) FOXM1 expression, score, and the proportion of high FOXM1 expression in patients’ tissue sections from non-metastatic or metastatic hepatoblastoma. mRNAsi, mRNA expression-based stemness index; WGCNA, weighted gene co-expression network analysis; FOXM1, forkhead box protein M1. ROC, receiver operating characteristic curve; AUC area under the curve. Data are shown as mean ± standard deviation (SD). * *p* < 0.05, ** *p* < 0.01, *** *p* < 0.001 and **** *p* < 0.0001.

The mRNAsi across the datasets (GSE133039, GSE131329, and GSE75271) exceeded 0.5 in a substantial majority of patients, which indicated a prevalent high-stemness phenotype within the cohorts (Figure 1a). Then, we performed weighted gene co-expression network analysis (WGCNA) to identify gene modules positively correlated with mRNAsi, of which those showing high correlation were prioritized for further analysis, such as the blue module in GSE133039 (Figure 1b,c), the red module in GSE131329 (Figure S1), and the brown module in GSE75271 (Figure S1). By taking the intersection of three key modules, we identified 102 hub stemness-related genes (Figure 1d and Table S1).

Functional enrichment analysis was subsequently conducted. Gene Ontology (GO) analysis revealed that these hub genes were predominantly involved in biological processes including cell cycle, mitosis, microtubule movement, DNA damage and repair, and ubiquitination (Figure 1e). The Reactome pathway analysis showed significant enrichment in cell cycle checkpoints and centromere signaling (Figure 1e). The concurrent activation of these pathways typically reflects robust cellular proliferation, DNA replication or genotoxic stress, particularly for tumor cells.

Transcription factors (TFs) were regarded as the pivotal regulators of tumor stemness and progression. The candidate TFs for the 102 hub genes were screened via the ChEA3 platform (Figure 1f), meanwhile we cross-referenced the result with the TRRUST database to identify the top 10 TFs ranked by q-value (Table S2). Notably, FOXM1 emerged as the sole transcription factor consistently identified by both CHEA3 and TRRUST (Figure 1g), which indicates FOXM1 as a potential crucial regulator of tumor stemness in hepatoblastoma.

### 2.2. FOXM1 Expression Increased in Metastatic Hepatoblastoma and the Clinical Correlation Analysis

FOXM1 expression was significantly higher in hepatoblastoma tissues compared to non-tumor liver tissues (Figure 1h). Receiver operating characteristic (ROC) curve analysis demonstrated that FOXM1 showed robust diagnostic performance, with area under the curve (AUC) values ranging from 0.793 to 0.970 (Figure 1i). Notably, FOXM1 expression was significantly elevated in hepatoblastoma patients with metastasis compared to those without metastasis (Figure 1j). The patients with longer overall survival or event-free survival exhibited significantly lower levels of FOXM1 expression (Figure 1k).

Among the 21 patients in our clinical center, 11 had metastatic disease and 10 did not. In the 11 patients with metastasis, all primary lesions showed high FOXM1 expression. Among the 10 patients without metastasis, 6 showed high FOXM1 expression and 4 showed low expression. A significantly higher proportion of metastatic patients was observed in the high-FOXM1-expression group (Figure 1l). Recurrent or metastatic lesions also showed high FOXM1 expression. The detailed clinical characteristics of these patients are summarized in Table S3.

Collectively, these results suggest that a high expression of FOXM1 is significantly associated with poor clinical outcomes.

### 2.3. FOXM1 Promoted Hepatoblastoma Cell Cycle and Proliferation In Vitro

**Figure 2:**
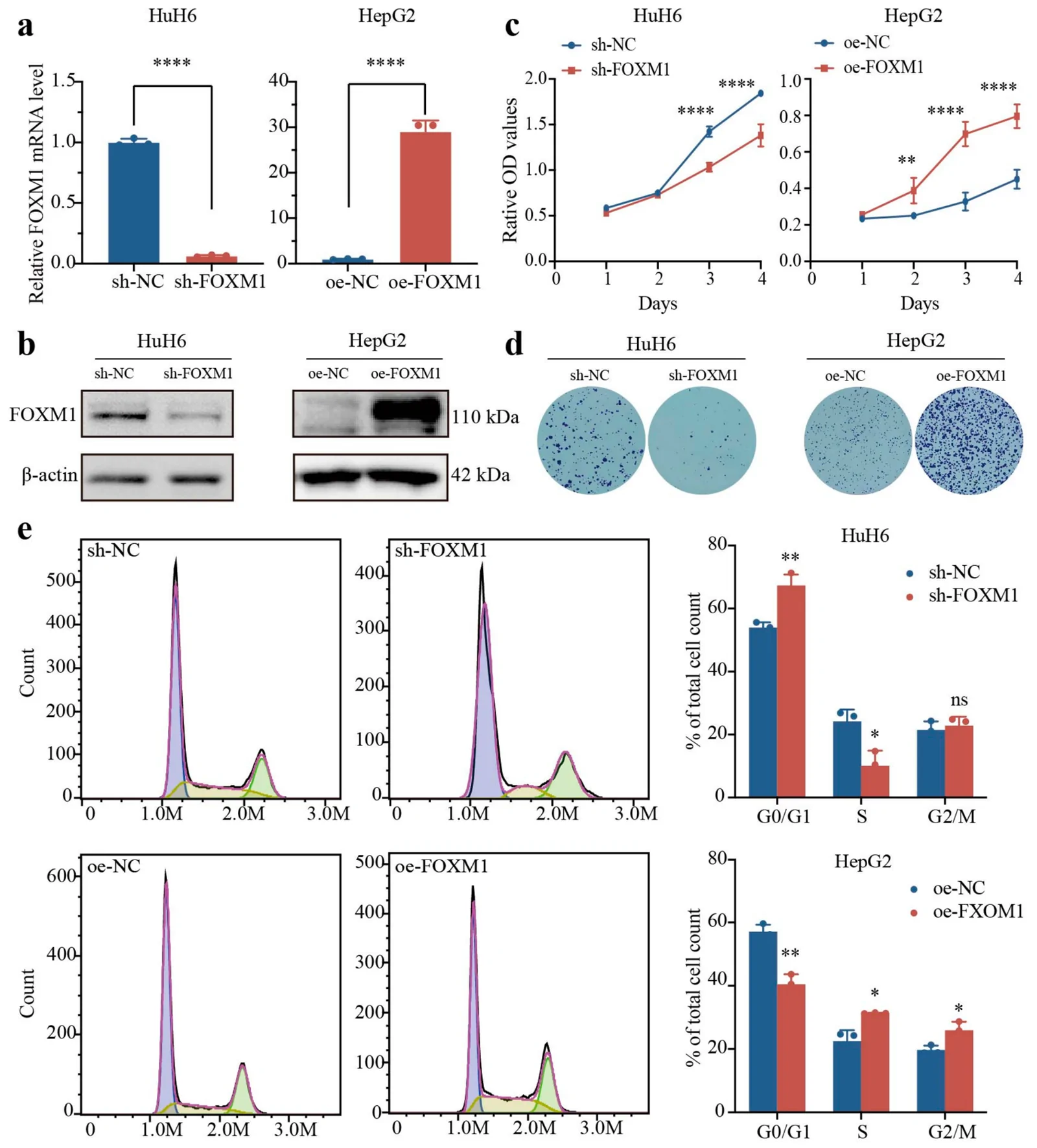
FOXM1 promoted hepatoblastoma cell cycle progression and proliferation in vitro. The efficiency of FOXM1 knockdown and overexpression was verified by RT-qPCR (**a**) and western blot (**b**). Cell proliferation was assessed by CCK-8 (**c**) and colony-formation assays (**d**) in HuH6 and HepG2 cells. (**e**) Cell cycle was analyzed by flow cytometry with propidium iodide staining. Data are shown as mean ± standard deviation (SD). *n* = 3 independent biological replicates, except for (**c**) (*n* = 4). * *p* < 0.05, ** *p* < 0.01, and **** *p* < 0.0001; ‘ns’ indicates no significant difference.

To investigate the biological function of FOXM1 in hepatoblastoma, we established stable FOXM1-knockdown HuH6 and FOXM1-overexpressing HepG2 cell lines, which were validated at both mRNA and protein levels (Figure 2a,b). The CCK-8 and colony formation assays demonstrated that FOXM1 knockdown significantly inhibited HuH6 cells’ proliferation, while conversely, FOXM1 overexpression markedly enhanced HepG2 cells’ proliferation compared to their respective control groups (Figure 2c,d). Then, we further examined how FOXM1 impacts cell cycle using flow cytometry. In FOXM1 knockdown cells, the proportion of cells in the G0/G1 phase was significantly increased, while the S phase population decreased (Figure 2e). This pattern indicates that FOXM1 knockdown induced G1/S cell cycle arrest. In contrast, FOXM1 overexpression led to a significant reduction in G0/G1 phase population, while both the S and G2/M phase populations significantly increased (Figure 2e). These findings suggest that elevated FOXM1 expression accelerated the G1-to-S transition and the progression of G2/M phase. Taken together, FOXM1 likely plays a critical role in promoting hepatoblastoma progression by facilitating cell cycle progression and cell proliferation.

### 2.4. FOXM1 Energized the Stemness of Hepatoblastoma Cells

**Figure 3:**
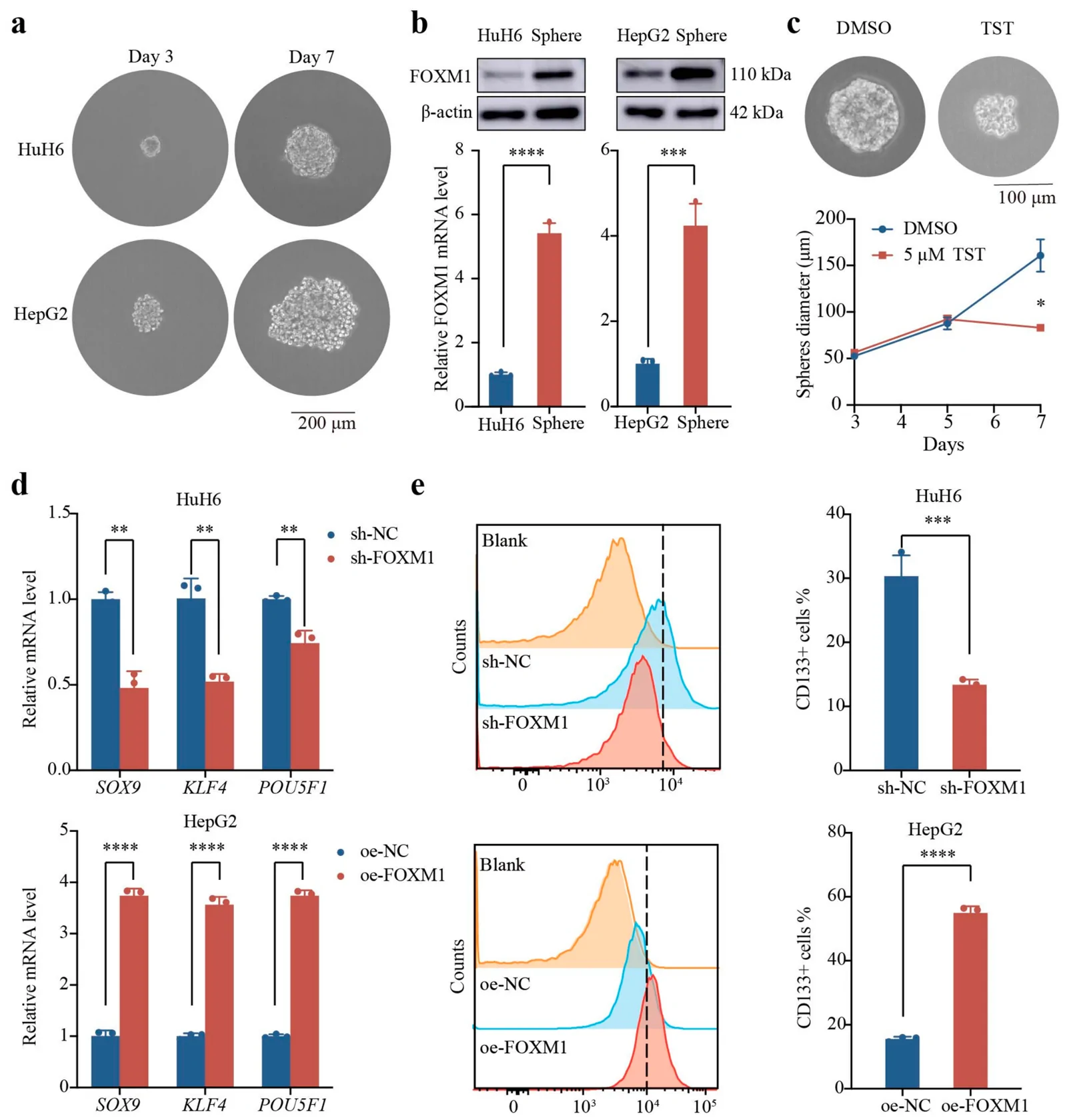
FOXM1 energized the stemness of hepatoblastoma cells. Effects of FOXM1 knockdown and overexpression on the stem cell-like phenotype of hepatoblastoma cells: (**a**) Representative images of tumor spheres formed from HuH6 and HepG2 cells at day 7, where HuH6-derived spheres appeared round and compact, whereas HepG2-derived spheres were relatively looser and less organized. (**b**) The mRNA and protein expression of FOXM1 in tumor spheres compared with the adherent parental cells. (**c**) Effect of TST on sphere size and morphology. (**d**) Expression of stemness-related genes *SOX9*, *KLF4* and *POU5F1* with FOXM1 knockdown and overexpression. (**e**) Percentage of CD133-positive cells, which is a key indicator of the cancer stem cell population. Data are shown as mean ± standard deviation (SD). *n* = 3 independent biological replicates. * *p* < 0.05, ** *p* < 0.01, *** *p* < 0.001 and **** *p* < 0.0001; TST, thiostrepton; DMSO, dimethyl sulfoxide.

Given the lack of widely accepted specific biomarkers for the CSCs in hepatoblastoma, current evaluations primarily rely on stemness gene expression, the proportion of CD133-positive cells, and sphere formation assays, which reflect stem-like features. Therefore, we utilized these established metrics to assess how FOXM1 affects hepatoblastoma cell stemness. Tumor spheres were generated from HuH6 and HepG2 cells and observed after 7 days of culture. Morphologically, HuH6-derived spheres appeared round and compact, whereas HepG2-derived spheres were relatively looser and less organized (Figure 3a). Notably, both the mRNA and protein levels of FOXM1 were significantly elevated in tumor spheres compared to the adherent parental cells (Figure 3b). At day 3 of culture, when treated with thiostrepton (TST), a FOXM1 inhibitor, sphere growth was markedly suppressed. Compared with the control, TST-treated spheres exhibited a significant reduction in diameter and developed a loose, irregular morphology (Figure 3c). Furthermore, we examined the expression of stemness-related genes. FOXM1 knockdown significantly decreased the expressions of SOX9, KLF4 and POU5F1, whereas FOXM1 overexpression markedly enhanced their levels (Figure 3d). Consistently, the proportion of CD133-positive cells, a key indicator of the cancer stem cell population, showed a similar trend (Figure 3e). Taken together, these findings suggest that FOXM1 plays a pivotal role in maintaining the stem cell-like phenotype of hepatoblastoma cells.

### 2.5. FOXM1 Facilitated Hepatoblastoma Metastasis by Inducing Cell Migration, Invasion and Epithelial–Mesenchymal Transition

**Figure 4:**
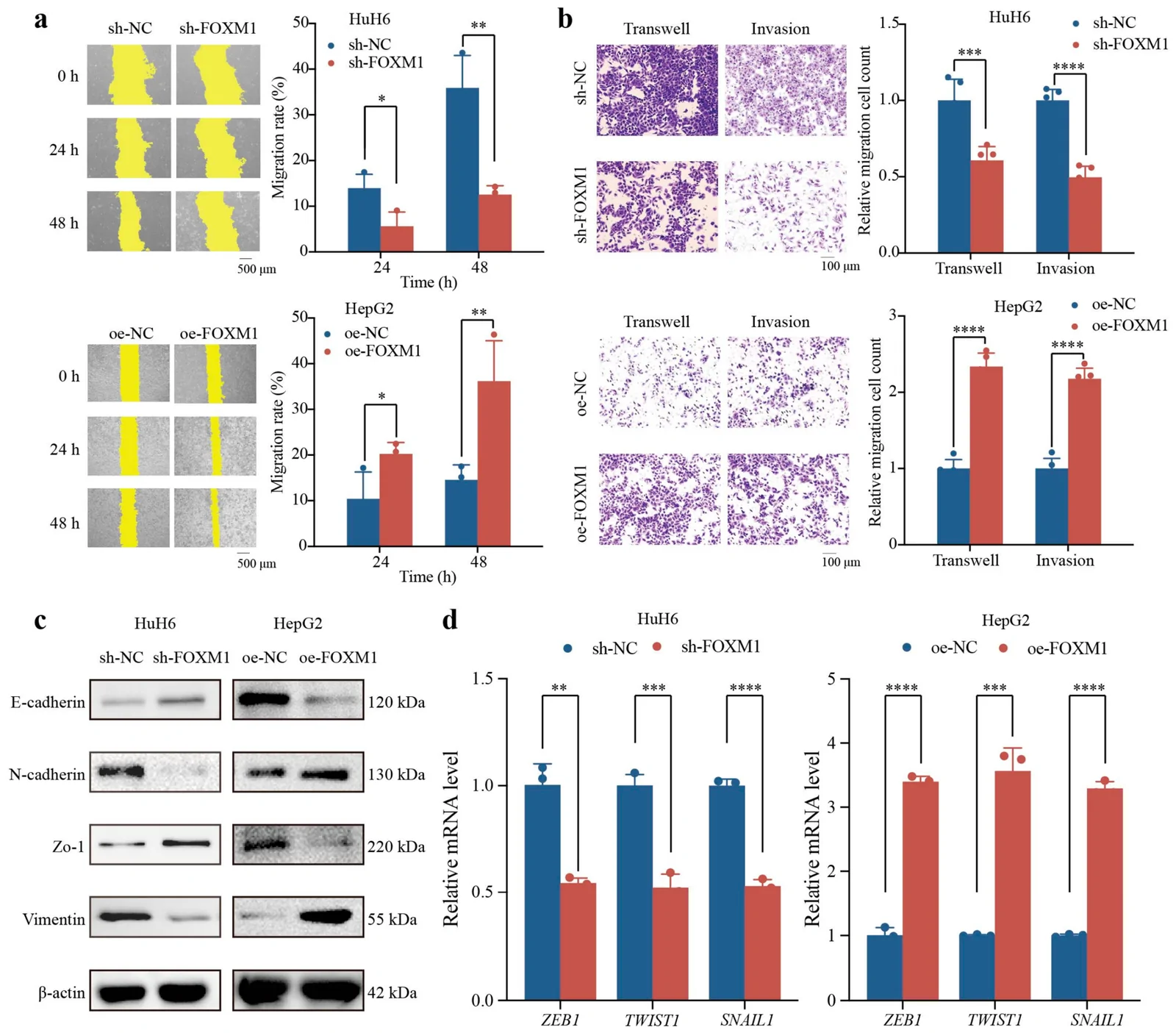
FOXM1 facilitated hepatoblastoma metastasis by inducing cell migration, invasion and epithelial–mesenchymal transition. Effects of FOXM1 knockdown and overexpression on hepatoblastoma cell migration, invasion and EMT: (**a**,**b**) The wound healing assays, transwell migration and matrigel invasion assays. (**c**) The expression of epithelial markers (E-cadherin and ZO-1) and mesenchymal markers (N-cadherin and Vimentin) by western blot. (**d**) RT-qPCR analysis of the EMT-associated transcription factors *ZEB1*, *TWIST*, and *SNAIL1*. Data are shown as mean ± standard deviation (SD). *n* = 3 independent biological replicates. * *p* < 0.05, ** *p* < 0.01, *** *p* < 0.001 and **** *p* < 0.0001.

The activation of epithelial–mesenchymal transition (EMT) can endow differentiated tumor cells with stemness traits [16], and has been widely regarded as playing important roles in tumor cell migration and invasion. Particularly, in hepatoblastoma, metastatic tumor cells have been shown to frequently exhibit an enriched stemness signature [10]. Therefore, we performed wound healing and transwell assays, which demonstrated FOXM1 knockdown significantly reduced wound closure and migrated cell numbers, as did TST treatment (Figure S2), whereas FOXM1 overexpression led to the opposite effect (Figure 4a). Consistently, the matrigel invasion assays revealed that FOXM1 knockdown impaired hepatoblastoma cell invasion, while its upregulation significantly enhanced it (Figure 4b). Furthermore, the western blot analysis showed that FOXM1 knockdown led to a significantly increased expression of epithelial markers (E-cadherin and ZO-1) with a decrease in mesenchymal markers (N-cadherin and Vimentin) (Figure 4c), while FOXM1 overexpression promoted the mesenchymal phenotype. Meanwhile, the transcription factors (ZEB1, TWIST and SNAIL1), which were well known to induce EMT [17], were significantly downregulated upon FOXM1 knockdown and upregulated following FOXM1 overexpression (Figure 4d). All the above findings suggest that FOXM1 enhances the migratory and invasive machinery of hepatoblastoma cells, accompanied by EMT activation.

### 2.6. FOXM1 Targeted the Promoter of TRIM59 in Hepatoblastoma Metastasis

**Figure 5:**
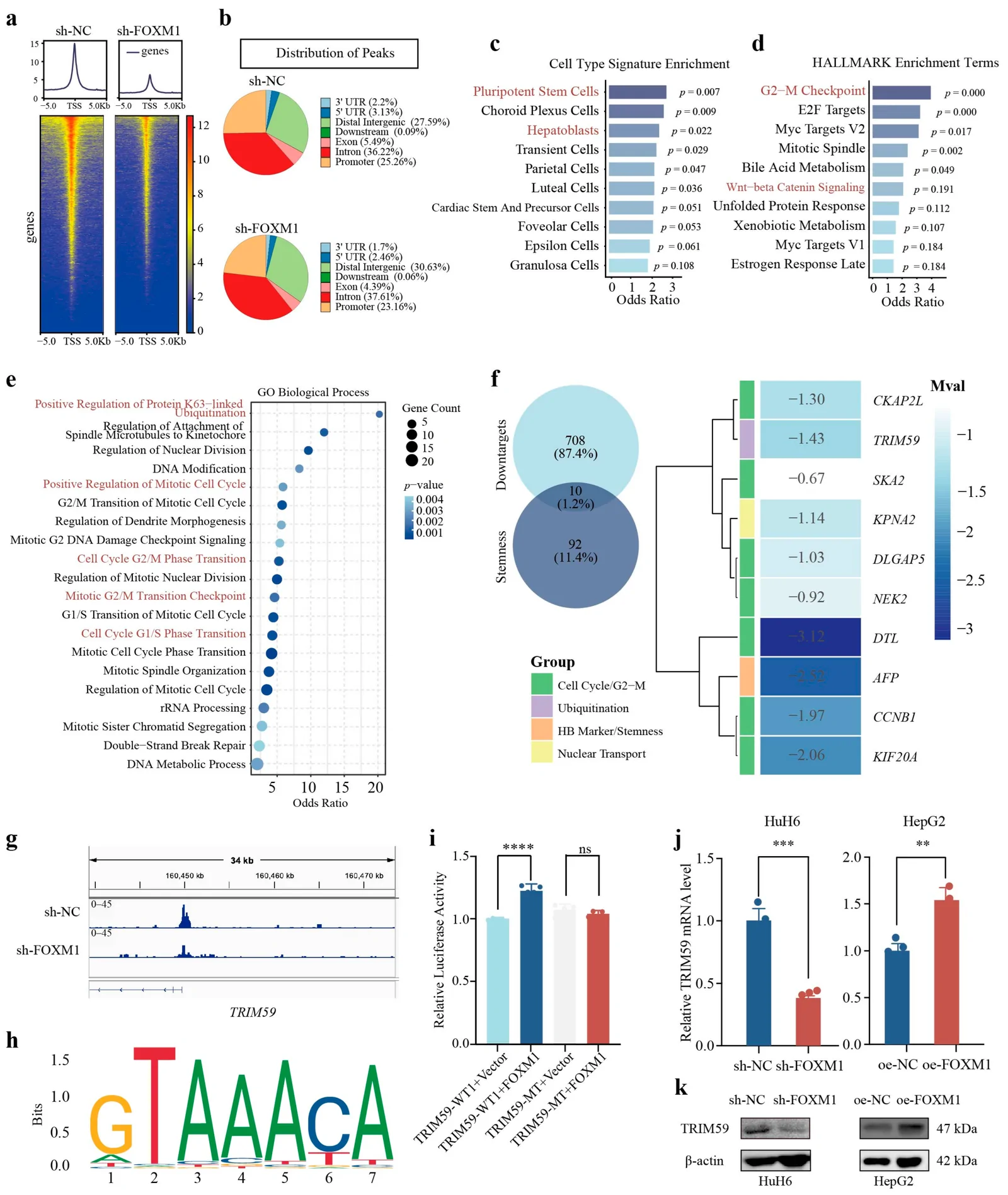
FOXM1 targeted the promoter of *TRIM59* in hepatoblastoma metastasis. (**a**) Based on CUT&Tag sequencing, the global distribution of FOXM1 binding sites significantly enriched around transcription start sites (TSS). (**b**) Genomic distribution analysis revealed that FOXM1 peaks localized to promoter regions (20–30%), distal intergenic regions, and introns. (**c**) Cell type enrichment analysis. (**d**) Hallmark enrichment (including Wnt/β-catenin signaling, *p* = 0.191) and (**e**) GO enrichment analysis. (**f**) Overlap between genes with reduced FOXM1 binding and stemness-related hub genes, including 10 genes involved in the cell cycle (*CKAP2L*, *SKA2*, *DLGAP5*, *NEK2*, *DTL*, *CCNB1*, *KIF20A*), ubiquitination regulation (*TRIM59*), the hepatoblastoma marker (*AFP*), and nuclear transport (*KPNA2*). (**g**) IGV tracks showed FOXM1 binding to the *TRIM59* promoter (M val = −1.43, M val.se = 0.30, *p* = 2.29 × 10^−6^, adjusted *p* = 0.011). (**h**) The predicted FOXM1 binding motif on the *TRIM59* promoter. (**i**) Dual-luciferase reporter assay. (**j**,**k**) The mRNA and protein expression of TRIM59. IGV, Integrative Genomics Viewer. Data are shown as mean ± standard deviation (SD). *n* = 3 independent biological replicates, except for (**i**) (*n* = 5). ** *p* < 0.01, *** *p* < 0.001 and **** *p* < 0.0001; ‘ns’ indicates no significant difference.

To further investigate the transcriptional regulatory mechanism of FOXM1 in hepatoblastoma metastasis, we performed CUT&Tag sequencing. The global distribution of FOXM1 binding sites showed significant enrichment around transcription start sites (TSS) (Figure 5a). Genomic distribution analysis revealed that FOXM1 peaks were predominantly localized to promoter regions (20–30%), distal intergenic regions, and introns (Figure 5b). The cell type enrichment analysis of the genes with reduced FOXM1 binding identified pluripotent stem cells and hepatoblasts as the top-ranked populations (Figure 5c). Notably, TFs enrichment analysis independently identified FOXM1 as the top-ranked transcription factor, validating the reliability of our sequencing data (Figure S3a). Hallmark enrichment analysis and GO analysis indicated that FOXM1-targeted genes were predominantly involved in the cell cycle (G1/S and G2/M transitions), ubiquitination regulation, and mitosis (Figure 5d,e and Figure S3b,c), mirroring the functional profiles of the previously identified hub genes. Although Wnt/β-catenin signaling was not significantly enriched in this analysis (*p* = 0.191, Figure 5d), we experimentally validated its functional activation based on clues from the previous literature [12,18,19].

By intersecting the hub genes with the reduced FOXM1 binding genes, we identified 10 core genes involved in the cell cycle (*CKAP2L*, *SKA2*, *DLGAP5*, *NEK2*, *DTL*, *CCNB1*, *KIF20A*), ubiquitination (*TRIM59*), the hepatoblastoma marker (*AFP*), and nuclear transport (*KPNA2*) (Figure 5f). Integrative Genomics Viewer (IGV) tracks demonstrated a significant reduction in FOXM1 binding peaks at the *TRIM59* promoter region when FOXM1 was knocked down (Figure 5g). Quantitative analysis revealed an approximately 2.69-fold decrease in FOXM1 binding intensity (Mval = −1.43, adjusted *p* = 0.011), indicating reduced FOXM1 chromatin occupancy at this site. Furthermore, we predicted the FOXM1-binding motif on the *TRIM59* promoter (Figure 5h) and performed dual-luciferase reporter assays. In 293T cells, the co-transfection of *FOXM1* vector and *TRIM59*-WT reporter significantly increased luciferase activity, whereas no difference was observed in the group with *TRIM59*-MT reporter (Figure 5i). FOXM1 knockdown led to a significant decrease in both TRIM59 mRNA and protein levels, while FOXM1 overexpression resulted in a marked upregulation of TRIM59 (Figure 5j,k). These results demonstrate that FOXM1 transcriptionally activated *TRIM59* in hepatoblastoma.

Finally, we performed correlation analysis between *TRIM59*, *FOXM1*, and mRNAsi. *TRIM59* expression exhibited a significant positive correlation with the mRNAsi (R = 0.36–0.68, *p* < 0.05; Figure S3d). More importantly, a robust correlation was observed between *FOXM1* and *TRIM59* mRNA levels (R = 0.51–0.83, *p* < 0.05; Figure S3e). These results from independent clinical datasets further reinforce the biological relevance of the FOXM1/TRIM59 axis in hepatoblastoma metastasis.

### 2.7. FOXM1 Accelerated Hepatoblastoma Metastasis by Targeting TRIM59 to Activate Wnt/β-Catenin Signaling Pathway

**Figure 6:**
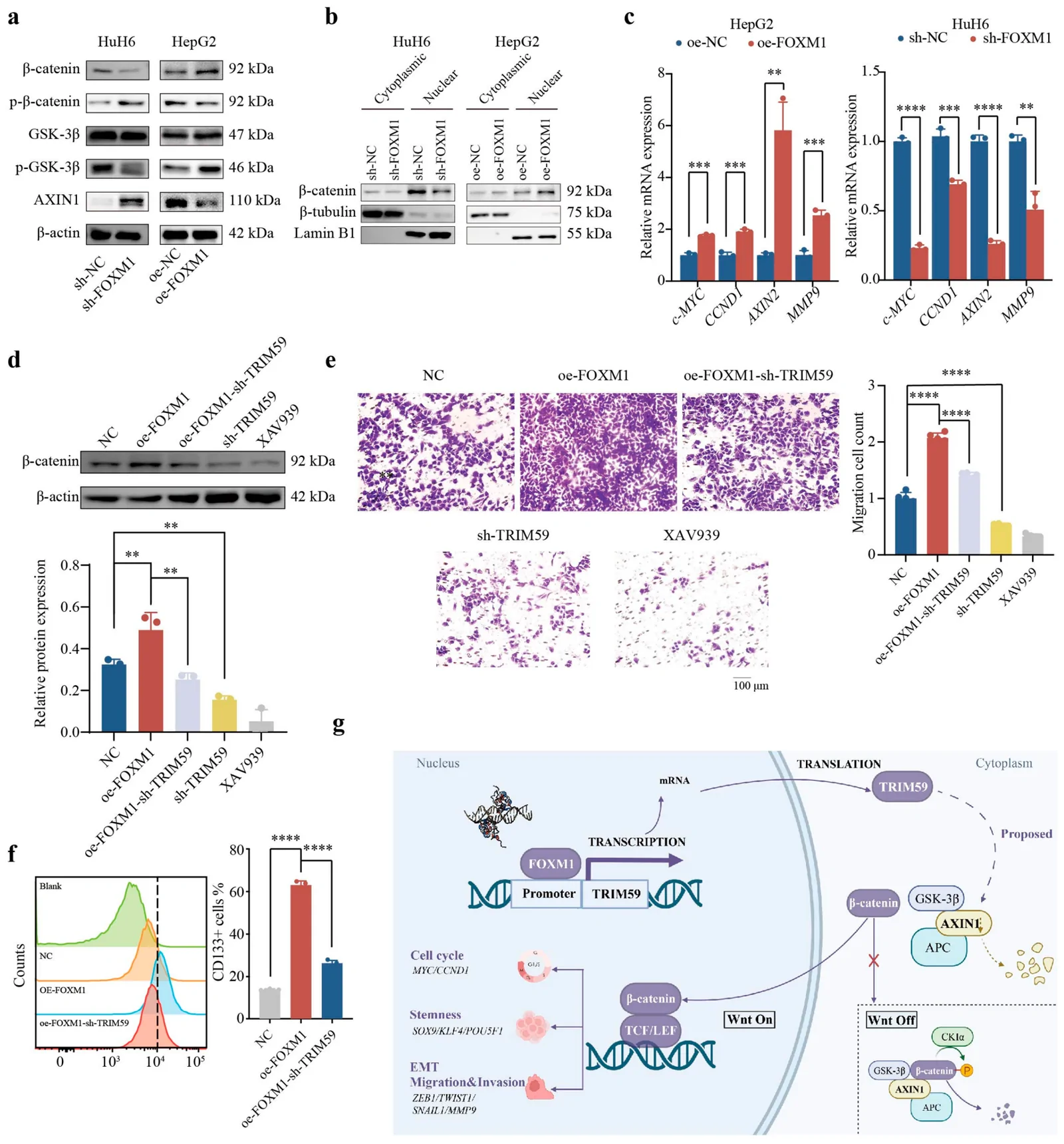
FOXM1/TRIM59/Wnt axis promotes hepatoblastoma stemness and metastasis. (**a**–**c**) Modulation of FOXM1 regulates Wnt pathway activity, showing (**a**) protein levels of Wnt/β-catenin pathway components (β-catenin, p-β-catenin, GSK-3β, p-GSK-3β, and AXIN1), (**b**) nuclear translocation of β-catenin, and (**c**) mRNA expression of downstream target genes (*c-Myc*, *CCND1*, *Axin2*, and *MMP9*). (**d**–**f**) Rescue experiments demonstrating the dependency on TRIM59 and Wnt signaling, illustrating (**d**) β-catenin protein levels, (**e**) migratory capacity and (**f**) CD133-positive cell proportions. (**g**) Proposed working model illustrating how FOXM1 sustains hepatoblastoma stemness and metastasis via the TRIM59/Wnt/β-catenin axis. The dashed line indicates the hypothesized regulatory link between TRIM59 and AXIN1, which requires future biochemical validation (created with BioRender.com). Data are shown as mean ± standard deviation (SD). *n* = 3 independent biological replicates. ** *p* < 0.01, *** *p* < 0.001 and **** *p* < 0.0001.

To elucidate the signaling cascade downstream of the FOXM1/TRIM59, we focused on the Wnt/β-catenin pathway, which is recognized as the core regulator of tumor stemness [10,18]. We first observed that FOXM1 significantly promoted the accumulation and nuclear localization of β-catenin, accompanied by alterations in the “destruction complex”, as evidenced by elevated p-GSK-3β and decreased AXIN1 levels (Figure 6a,b). These changes were associated with the activation of canonical Wnt target genes, such as *c-My*c, *CCND1*, and *MMP9* (Figure 6c). Notably, MMP9 facilitates tumor dissemination by degrading the extracellular matrix to create a permissive environment for cell motility [20].

We then performed rescue experiments to determine whether TRIM59 was the critical link between FOXM1 and Wnt activation. Notably, TRIM59 knockdown effectively reversed the FOXM1-overexpression-induced increase in β-catenin levels and the expansion of CD133-positive cells induced by FOXM1 overexpression (Figure 6d,f). Consistently, the enhanced cell migration induced by FOXM1 overexpression was also significantly diminished upon TRIM59 knockdown (Figure 6e). In addition, treatment with the Wnt inhibitor XAV939 alone reduced β-catenin levels and impaired migratory capacity, demonstrating that Wnt/β-catenin signaling is functionally required for maintaining these properties in hepatoblastoma cells (Figure 6d). Collectively, these findings suggest that FOXM1 promoted hepatoblastoma metastasis by targeting *TRIM59* to activate the Wnt/β-catenin signaling pathway, via enhancing the β-catenin accumulation and nuclear translocation (Figure 6g).

### 2.8. FOXM1 Stimulated Hepatoblastoma Tumor Growth and Experimental Lung Metastasis In Vivo

**Figure 7:**
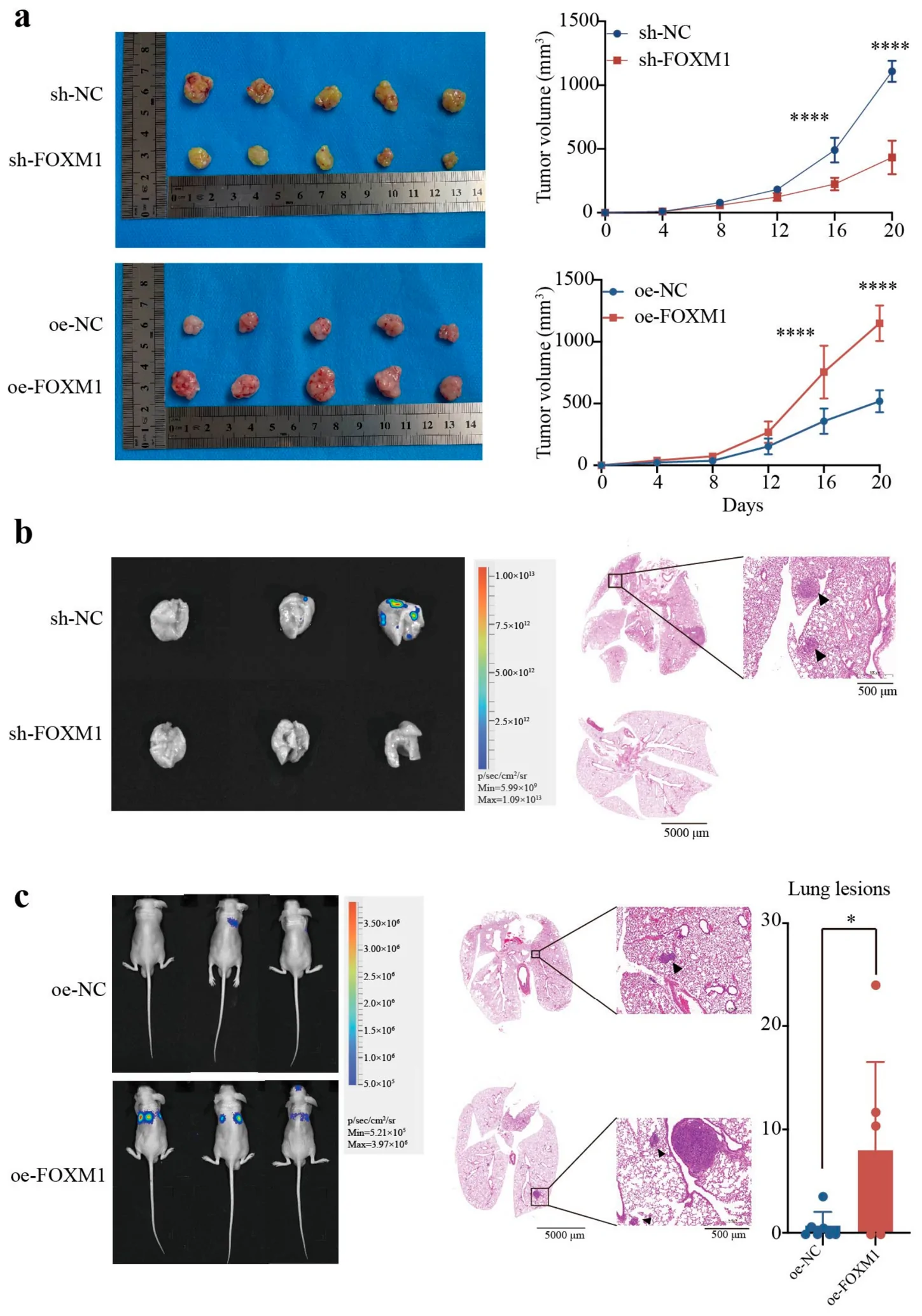
FOXM1 drives hepatoblastoma growth and experimental lung metastasis in vivo. (**a**) Images and statistical analysis (volume) subcutaneous xenograft tumors formed by sh-NC/sh-FOXM1 HuH-6 cells and oe-NC/oe-FOXM1 HepG2 cells. (**b**) Fluorescence images and histological HE-stained lung sections from the sh-NC and sh-FOXM1 groups; black arrowheads denote metastatic nodules. (**c**) Bioluminescence images, corresponding HE-stained lung sections, and total lung lesion areas from the oe-NC and oe-FOXM1 groups. Data are shown as mean ± standard deviation (SD). *n* = 7 independent biological replicates. * *p* < 0.05, **** *p* < 0.0001.

Subsequently, we established a subcutaneous xenograft model in nude mice using FOXM1-knockdown HuH6 and FOXM1-overexpressing HepG2 cell lines. FOXM1 knockdown significantly retarded hepatoblastoma expansion, as evidenced by the reduced tumor volumes compared to control, whereas FOXM1 overexpression markedly accelerated tumor growth (Figure 7a).

We further evaluated a tail-vein injection model. Green fluorescent protein (GFP) imaging demonstrated that lung colonization was detectable in two out of seven mice in the sh-NC group, while there was no lung colonization in the sh-FOXM1 group (Figure 7b). Conversely, in a gain-of-function model, bioluminescence imaging revealed that FOXM1 overexpression increased the incidence of experimental metastasis (five out of seven in the oe-FOXM1 group vs. three out of seven in the oe-NC group) (Figure 7c). Although neither comparison of metastatic incidence reached statistical significance, they showed a trend consistent with the in vitro migration and invasion results. Importantly, HE-stained lung sections confirmed that the total area of lung colonization was significantly larger in the FOXM1-overexpressing group (Figure 7c). The raw area measurements for every individual animal are detailed in Table S4. Collectively, these results support FOXM1 as a critical driver of hepatoblastoma growth and metastasis, providing potential targets for clinical translational therapy.

## 3. Discussion

Approximately 20% of hepatoblastoma (HB) patients present with metastasis, which is associated with a dismal prognosis [21]. A series of studies have suggested that tumor stemness is a key driver of metastasis and recurrence [7,8,9], most consistently in hepatoblastoma, in which the canonical Wnt/β-catenin pathway is frequently dysregulated [11,12]. Aberrant Wnt/β-catenin signaling in hepatoblastoma is mostly initiated by somatic mutations in exon 3 of CTNNB1 that lead to the nuclear accumulation of β-catenin and subsequent promotion of metastatic potential [22,23]. However, the overall tumor mutational burden in hepatoblastoma is remarkably low, suggesting that metastasis seems to be driven more by epigenetic and post-transcriptional dysregulation [24,25]. In this study, the post-translational regulation of protein stability, as an alternative mechanism in the “mutation-poor” environment, may play a role in hepatoblastoma metastasis. Precisely, by bridging bioinformatics with in vivo and in vitro experiments, we demonstrate that FOXM1 transcriptionally upregulates TRIM59, which acts as a functional upstream regulator of Wnt/β-catenin signaling to drive stemness, migration, invasion, and EMT in hepatoblastoma. Although the precise biochemical mechanism linking TRIM59 to the Wnt destruction complex requires further elucidation, this FOXM1/TRIM59/Wnt axis offers new insights into the mechanistic basis of hepatoblastoma and potential therapeutic targets.

FOXM1 is a well-known transcription factor that orchestrates cancer progression and metastasis [14]. FOXM1 often works through tissue-specific axes, such as FOXM1/Smad4 positive feedback loop in pancreatic cancer [26], the FOXM1/CEBPB axis in hepatocellular carcinoma [27], and AXL/eEF2 signaling in glioblastoma multiforme [28], by modulating developmental heterogeneity, immune surveillance, antitumor immunity and ferroptosis. In the current study, we demonstrated a significant up-regulation of FOXM1 in both hepatoblastoma patient tissues and hepatoblastoma-derived tumor spheres, which effectively facilitated the core malignant tumor hallmarks, including proliferation, stemness, EMT, migration and invasion. The tumor stemness and EMT represent two critical facets of cellular plasticity that collectively orchestrate the metastatic cascade [29]. Our results reveal that FOXM1 knockdown not only reduced stem-like features, but also effectively impaired the EMT and hepatoblastoma cell migration. This synergy aligns with the established paradigm that metastatic hepatoblastoma cells frequently exhibit an enriched stemness signature [10].

TRIM family proteins are emerging as significant biomarkers for cancer diagnosis and prognosis due to extensive evidence for their crucial roles in chemoresistance, EMT, and the CSC phenotypes [30,31]. Particularly, the E3 ubiquitin ligase TRIM59 has been shown to contribute to various malignancies [15,32,33,34]. Here, we identified TRIM59 as a key target gene of FOXM1 in hepatoblastoma. The FOXM1 overexpression and subsequent TRIM59 increase were accompanied by a significant reduction in AXIN1 and decreased p-β-catenin (Ser33/37/Thr41, the phosphorylated form destined for degradation), as well as a marked elevation in p-GSK-3β (Ser9, normally represents the inactive state of the kinase). These findings suggest that the FOXM1/TRIM59 axis may affect AXIN1 protein levels, which is the rate-limiting scaffold of the destruction complex [35]. Reduced AXIN1 levels may lead to the decreased phosphorylation and degradation of β-catenin, potentially facilitating β-catenin nuclear translocation and the subsequent activation of downstream targets (*MMP9*, *c-MYC*, etc). Functionally, our rescue experiments demonstrated that TRIM59 knockdown effectively reversed FOXM1-induced β-catenin accumulation and malignant phenotypes, supporting the functional dependency of Wnt/β-catenin signaling on TRIM59. At the molecular level, based on these observations, we hypothesize that TRIM59 may promote AXIN1 degradation, potentially through ubiquitination, thereby relieving the destruction complex’s inhibition of β-catenin. This hypothesis is supported by a recent study in the mouse spermatogonial cell line GC-1, which demonstrated that TRIM59 directly ubiquitinates AXIN1, leading to the activation of β-catenin signaling, and that β-catenin activation reverses the effects of TRIM59 knockdown [36]. Although whether this exact mechanism operates in hepatoblastoma remains to be determined, this precedent reinforces the plausibility of the TRIM59/AXIN1/β-catenin axis in our model. Consistent with our study, recent research also highlighted the role of ubiquitination in hepatoblastoma, noting a significant down-regulation of TRIM29 in metastatic hepatoblastoma cell lines [18]. By identifying AXIN1 as a potential downstream target of TRIM59 E3 ligase and proposing the FOXM1–TRIM59–Wnt axis as a regulatory mechanism, our study provides a possible explanation for the constitutive activation of Wnt/β-catenin signaling in metastatic hepatoblastoma. Importantly, this axis appears to operate in a *CTNNB1*-mutant context and amplify β-catenin signaling.

The FOXM1/TRIM59 axis seems valuable in clinical translation for metastatic hepatoblastoma early diagnosis and treatment, and it has been highlighted in emerging pharmacological strategies targeting cancer cell plasticity. For instance, the small-molecule FOXM1 inhibitor thiostrepton has demonstrated potent anti-tumor activity in various preclinical cancer models [37,38,39]. Recent advances in RNAi-based therapeutics suggest a promising blueprint for the clinical intervention of hepatocellular carcinoma [27]. Similarly, this study has shown that thiostrepton inhibited spheroid formation in hepatoblastoma cells and reduced metastatic potential. Taken together, these findings underscore the need for a deeper mechanistic understanding, and provide promising targeted therapeutic strategies.

Despite this, several areas warrant further investigation. Firstly, our study provides evidence for FOXM1/TRIM59 as new candidate molecules to identify and target metastatic hepatoblastoma; however, they still require validation in large-scale, multi-center clinical cohorts. Secondly, serum starvation cannot completely exclude the contribution of proliferation over the 48-h assay period. Nevertheless, the robust induction of EMT and MMP9 strongly supports that FOXM1 intrinsically enhances the migratory machinery. Thirdly, self-renewal capacity was not formally assessed; sphere formation, CD133 positivity, and stemness-related gene expression reflect stem-like features rather than self-renewal, and serial passaging sphere formation or limiting dilution transplantation assays were not performed. Fourthly, the direct physical interaction between TRIM59 and AXIN1, as well as TRIM59-mediated AXIN1 ubiquitination, remain unproven in our study and warrant future biochemical validation. Future studies will be essential to elucidating this post-translational mechanism. Finally, the tail-vein injection model primarily assesses lung colonization rather than spontaneous metastasis from a primary liver tumor. Orthotopic liver tumor models would be required to evaluate the full metastatic cascade. These limitations should be considered when interpreting our findings, and future studies are needed to address them.

## 4. Materials and Methods

### 4.1. The Genome Data and Preprocessing

The bulk transcriptomic datasets (GSE151347, GSE133039, GSE131329, and GSE75271) were downloaded from the Gene Expression Omnibus (GEO) database (Table S5). All datasets underwent rigorous quality control and normalization prior to downstream analysis. Specifically, the RNA sequencing data were normalized using the variance stabilizing transformation (VST) function of the DESeq2 package (v1.38.3), while the microarray data were subjected to quantile normalization via the normalizeBetweenArrays() function of the limma package (v3.54.2).

### 4.2. Calculation of mRNA Expression-Based Stemness Index

The mRNA expression-based stemness index (mRNAsi) was calculated to quantify the oncogenic dedifferentiation. The mRNAsi ranges from 0 to 1, where higher values correspond to more pronounced stemness characteristics. Following the methodology set out by Malta et al. [40], the stem cell data from the Progenitor Cell Biology Consortium (PCBC) were utilized as a training set. A predictive model was constructed using the one-class logistic regression (OCLR) machine learning algorithm via the gelnet package (v1.2.1) to calculate the mRNAsi for each sample.

### 4.3. Weighted Correlation Network Analysis

Weighted gene co-expression network analysis (WGCNA) was performed to identify gene modules highly correlated with tumor stemness. Each module was summarized by the module eigengenes and used for subsequent correlation analysis with clinical and phenotypic traits. In this study, modules strongly associated with mRNAsi were identified. Three datasets (GSE133039, GSE131329, and GSE75271) met the sample size threshold for WGCNA, while GSE151347 was excluded due to an insufficient sample size (*n* < 15) [41].

### 4.4. Identification of Hub Stemness-Related Genes and Regulatory Networks

The intersection of genes identified from the three datasets was defined as the hub stemness-related genes. Subsequently, functional enrichment analysis, including Gene Ontology (GO) and Reactome pathways, was conducted. Transcription factors (TFs) that regulate the hub genes were screened in the ChEA3 and TRRUST databases, followed by a transcriptional regulatory network analysis. Furthermore, we performed correlation analysis, receiver operating characteristic curve (ROC) analysis and differences expression analysis to validate the robust association between the key transcription factor FOXM1 and clinical outcomes in hepatoblastoma datasets.

### 4.5. Hepatoblastoma Sample and Immunohistochemistry

A total of 27 tissue blocks from 21 hepatoblastoma patients (2018–2023) were fixed, paraffin-embedded, and cut at 4 μm. Antigen retrieval was performed in citrate buffer (pH 6.0, pressure, 40 min). After blocking, sections were incubated with anti-FOXM1 (Santa Cruz, Dallas, TX, USA, Cat# sc-376471, 1:500) overnight, followed by biotinylated secondary and HRP-conjugated tertiary antibodies (50 min each). DAB was used for visualization, with hematoxylin counterstaining. The staining index (SI), which was calculated by multiplying the score for staining intensity and the percentage of positive tumor cells, was used for semi-quantitative evaluation. Staining intensity was graded on a four-point scale: 1 (no staining), 2 (weak; light yellow), 3 (moderate; yellow-brown), and 4 (strong; brown). The percentage of positive tumor cells was scored as follows: 0 (no positive cells), 1 (<10%), 2 (10–35%), 3 (35–75%), and 4 (>75%). The SI values range was 0, 1, 2, 3, 4, 6, 8, 9, 12, or 16, and a score > 6 was defined as high expression in subsequent analysis [42,43]. Immunohistochemical scoring was performed while blinded to the clinical data.

### 4.6. Cell Culture and Lentiviral Infection

The HuH6 and HepG2 cell lines were purchased from BAIDI Biotechnology Ltd (Hangzhou, China). and cultured in Dulbecco’s modified Eagle’s medium (DMEM, Gibco, Grand Island, NY, USA) supplemented with 10% fetal bovine serum and 1% penicillin/streptomycin, at 37 °C in a humidified incubator with 5% CO_2_. Lentiviral vectors for FOXM1 knockdown (sh-FOXM1 with a GFP tag, purchased from GeneChem (Shanghai, China)), FOXM1 overexpression (oe-FOXM1 carrying a luciferase tag, purchased from GeneChem (Shanghai, China)), and TRIM59 knockdown (sh-TRIM59 without a tag, purchased from Hanbio (Shanghai, China)) were employed in accordance with the official guidebook instructions. The knockdown and overexpression efficiencies in HepG2 and HuH6 with the respective lentiviruses were validated by quantitative real-time PCR and western blotting.

### 4.7. Cell Proliferation and Colony Formation Assay

Cell viability was assessed using the Cell Counting Kit-8 (CCK-8; Beyotime, Shanghai, China). Hepatoblastoma cells were seeded into 96-well plates at a density of 5000 cells per well. At 0, 24, 48, and 72 h post-seeding, 10% CCK-8 reagent was added to each well, followed by incubation for 1 h at 37 °C. The absorbance at 450 nm was then measured using a microplate reader. For the colony formation assay, cells were seeded into 6-well plates at a density of 1000 cells per well and cultured for 10–14 days. The colonies were then washed with phosphate-buffered saline (PBS), fixed with 4% paraformaldehyde, and stained with 0.1% crystal violet for 15 min. The colonies (defined as clusters containing ≥50 cells) were counted and photographed.

### 4.8. Sphere Formation Assay

Hepatoblastoma cells were seeded at a density of 5 × 10^4^ cells into ultra-low attachment T25 flasks (LABSELECT, Hefei, China) containing DMEM/F12 medium supplemented with 20 ng/mL basic fibroblast growth factor, 20 ng/mL epidermal growth factor, and 2% B27 supplement. After 4–7 days, sphere formation was observed and imaged. Cell spheres were then treated with thiostrepton and images were captured at days 0, 4, and 7 days post-treatment; DMSO was used as the control.

### 4.9. Wound Healing Assay

Hepatoblastoma cells were seeded in 6-well plates. Upon reaching 90% confluence, the cells were starved in serum-free medium overnight, and a scratch was made in the cell monolayer using a sterile 200 μL pipette tip. Debris was removed by washing with PBS, and the medium was replaced with serum-free medium. Wound healing was imaged at 0, 24, and 48 h. The migration rate was quantified by measuring the wound area using ImageJ software (v2.14.0).

### 4.10. Cell Migration and Invasion Assays

Cell migration and invasion assays were performed using 24-well culture plates with 8 µm pore inserts. For migration assays, HepG2 (4 × 10^4^) or HuH6 (8 × 10^4^) cells in medium containing 1% fetal bovine serum (FBS) were seeded into the upper wells and incubated for 48 h. For invasion assays, the upper wells were pre-coated with 80 µL of Matrigel (Corning Inc., Corning, NY, USA, Cat# 356234) diluted 1:40 in serum-free medium. After 48 h of incubation, non-migrated cells were gently removed with a cotton swab, and the cells on the lower surface were fixed and stained with 0.1% crystal violet for 45 min.

### 4.11. Flow Cytometry Analysis of Cell Cycle and CD133 Expression

For cell cycle analysis, cells were fixed in ice-cold 75% ethanol at 4 °C overnight, and then stained with propidium iodide (PI). For CD133 expression analysis, 1 × 10^6^ cells were incubated with allophycocyanin (APC)-conjugated mouse anti-human CD133 antibody (BioLegend, San Diego, CA, USA, Cat# 393905) according to the manufacturer’s instructions. Stained cells were analyzed with a NovoCyte flow cytometer (Agilent Technologies, Santa Clara, CA, USA), followed by FlowJo (version 10.8.1) software to process data.

### 4.12. Real-Time Quantitative Polymerase Chain Reaction

Total RNA was extracted using TRIzol™ reagent, followed by a NanoDrop spectrophotometer to determine the purity and concentration. The cDNA was synthesized via the Reverse Transcription Kit (Mei5, Beijing, China, Cat# MF166). Subsequently, real-time quantitative polymerase chain reaction (RT-qPCR) was performed on an ABI 7300 system (Foster City, CA, USA) using SYBR Green master mix (Zoman, Beijing, China, Cat# ZF501-2). Each sample was subjected to three independent replicates. Relative gene expression was calculated using the 2^−ΔΔCt^ method, with β-actin as the internal control. The primer sequences are listed in Table S6.

### 4.13. Western Blotting

Cells were lysed using RIPA buffer, with protease and phosphatase inhibitors. Equal amounts of protein were separated by SDS-PAGE, transferred to PVDF membranes and blocked with 5% non-fat milk or 5% BSA, then incubated with primary and secondary antibodies. Protein bands were visualized using enhanced chemiluminescence (ECL).

Membranes were probed with antibodies against β-actin, β-tubulin, and Lamin B1 to confirm equal loading. Densitometric analysis was performed using ImageJ software (v2.14.0). The following primary antibodies were used: anti-FOXM1 (Proteintech, Wuhan, China, 1:1000); anti-TRIM59 (Proteintech, Wuhan, China, 1:1000); anti-E-cadherin (Proteintech, Wuhan, China, 1:50,000); anti-N-cadherin (Proteintech, Wuhan, China, 1:10,000); anti-Vimentin (Santa Cruz, Shanghai, China, 1:1000); anti-ZO-1 (Proteintech, Wuhan, China, 1:5000); anti-β-catenin (ImmunoWay, Suzhou, China, 1:5000); anti-phospho-β-catenin (ImmunoWay, Suzhou, China, 1:1000); anti-APC (ImmunoWay, Suzhou, China, 1:10,000); anti-GSK-3β (ImmunoWay, Suzhou, China, 1:1000); and anti-phospho-GSK-3β (ImmunoWay, Suzhou, China, 1:10,000). HRP-linked anti-IgG (ImmunoWay, Suzhou, China, 1:5000) was used as the secondary antibody.

### 4.14. CUT&Tag Assay and Data Analysis

The Cleavage Under Targets and Tagmentation (CUT&Tag) assay was performed using the Hyperactive Universal CUT&Tag Assay Kit for Illumina (Vazyme, Nanjing, China, Cat# TD904) according to the manufacturer’s instructions, with minor modifications as previously described [44,45]. Briefly, 1 × 10^5^ cells per sample were washed and incubated with activated Concanavalin A (ConA) magnetic beads (10 μL/sample) in Binding Buffer for 10 min at room temperature. After removing the supernatant, the cell–bead complexes were resuspended in pre-cooled Antibody Buffer and incubated with an anti-FOXM1 primary antibody (Proteintech, Cat# 13147-1-AP, 1 μg/sample) overnight at 4 °C. Following washes, samples were incubated with a goat anti-rabbit secondary antibody (1:100; Vazyme) for 1 h at room temperature. After washing with Dig-wash Buffer to remove unbound antibodies, the complexes were incubated with pA/G-Tnp Pro transposase (0.04 μM in Dig-300 Buffer) at room temperature for 1 h. After extensive washing with Dig-300 Buffer to remove unbound transposase, tagmentation was initiated by resuspending the complexes in reaction mix (Dig-300 Buffer and 5× TTBL) and incubating at 37 °C for 1 h. The reaction was terminated by adding 10% SDS and E. coli DNA Spike-in (Vazyme, 1 pg/100,000 cells) at 55 °C for 10 min. DNA was extracted using DNA Extract Beads Pro, and libraries were prepared via 13-cycle PCR using the provided Amplification Mix and TruePrep Index Kit V2 (Vazyme, Cat# TD202). The PCR products were then purified using VAHTS DNA Clean Beads (Vazyme, Cat# N411). Finally, libraries were sequenced by Novogene on the Illumina NovaSeq X Plus platform (PE150).

Raw sequencing reads were quality-trimmed and aligned to the human reference genome (GRCh38/hg38). Peaks with |Mval| > 0.57 and *p* < 0.05 were considered differentially bound, where Mval represents the log2-scaled difference in normalized read counts between conditions (Mval = 1 corresponds to a two-fold change) [46]. Peaks with positive and negative Mval were classified as increased and reduced FOXM1 binding, respectively.

### 4.15. Dual-Luciferase Assay

Dual-luciferase reporter assays were performed according to the manufacturer’s instructions (GeneCreate, Wuhan, China, Cat: JKR23008). The putative region of the *TRIM59* gene promoter where the transcription factor FOXM1 binds, either wild-type or mutant, was cloned upstream of the luciferase reporter gene. The resulting constructs were co-transfected into HEK293T cells alongside either the FOXM1 overexpression plasmid (pcDNA3.1-FOXM1) or the corresponding empty vector. After 48 h, cells were lysed and firefly luciferase activity was measured using the Dual-Luciferase Reporter Assay System, with Renilla luciferase activity serving as an internal control for normalization.

### 4.16. Animal Experiment

For the in vivo tumorigenicity assay, four-week-old male nude mice were obtained from Shaanxi Pharmaceutical Medical Biotechnology Co., Ltd. (Xi’an, China). A total of 5 × 10^6^ cells suspended in 100 μL serum-free medium were subcutaneously injected into the right flanks of mice (*n* = 7 per group). Tumor growth was monitored every 4 days by measuring tumor dimensions with a caliper. Tumor volume was calculated using the formula V = 0.5 × length × width^2^.

For the in vivo experimental lung metastasis assay, nude mice (*n* = 7 per group) received tail-vein injections of 2 × 10^6^ cells in 100 μL PBS. Specifically, HuH6 cells with stable FOXM1 knockdown (sh-FOXM1-GFP) and HepG2 cells with stable FOXM1 overexpression (oe-FOXM1-Luc), along with their respective negative controls, were utilized. Lung colonization was monitored 4–5 weeks post-injection via bioluminescence or fluorescence imaging using the Tanon ABL-X5 In Vivo Imaging System (Tanon, Shanghai, China). Upon sacrifice, lung tissues were harvested for hematoxylin and eosin (H&E) staining to quantify metastatic burden. Metastatic lesion burden was defined as the total lung lesion area (in pixels^2^) across two representative lung sections per mouse, which was quantified using ImageJ software (v2.14.0) [47].

All animal experiments were performed in accordance with the ARRIVE guidelines 2.0 [48].

### 4.17. Statistical Analysis

Data are shown as mean ± SD from three independent biological replicates, unless otherwise stated. Two-group comparisons were performed using a two-tailed Student’s t-test or Mann–Whitney U test. For multiple comparisons, Sidak’s test was used for repeated time-point measurements, and Dunnett’s test for multiple groups versus a single control. *p* < 0.05 was considered significant. Statistical analyses were conducted using R (v4.5.2) and GraphPad Prism (v10.2.1). Statistically significant difference was marked with asterisks: *, *p* < 0.05, **, *p* < 0.01, ***, *p* < 0.001 and ****, *p* < 0.0001; ‘ns’ indicates no significant difference.

## 5. Conclusions

In conclusion, our study demonstrates that FOXM1 transcriptionally regulates TRIM59 to promote hepatoblastoma metastasis, potentially mediated by the activation of Wnt/β-catenin signaling. This work not only provides new insights into the etiological mechanisms, but also highlights FOXM1 and TRIM59 as promising therapeutic targets for high-risk patients.

## Data Availability

The datasets analyzed in this study are publicly available in the GEO database. The data reported in this paper have been deposited in the OMIX, China National Center for Bioinformation/Beijing Institute of Genomics, Chinese Academy of Sciences (https://ngdc.cncb.ac.cn/omix: accession no. OMIX021513) (accessed on 4 October 2026).

## Supplementary Materials

The following supporting information can be downloaded at: https://www.mdpi.com/article/10.3390/ijms27219843/s1.

## Abbreviations

The following abbreviations are used in this manuscript:

APC	Allophycocyanin
AUC	Area Under The Curve
AXIN1	Axis Inhibition Protein 1
CCK-8	Cell Counting Kit-8
CSC	Cancer Stem Cell
CUT&Tag	The Cleavage Under Targets and Tagmentation assay
DMEM	Dulbecco’s modified Eagle’s medium
DMSO	Dimethyl Sulfoxide
EMT	epithelial-mesenchymal transition
FBS	Fetal Bovine Serum
FOXM1	Forkhead Box Protein M1
GEO	Gene Expression Omnibus
GFP	Green Fluorescent Protein
GO	Gene Ontology
GS	Gene Significance
H&E	Hematoxylin and Eosin
HB	Hepatoblastoma
IC_50_	50% Inhibitive Concentration
IGV	Integrative Genomics Viewer
MM	Module Membership
mRNAsi	mRNA Expression-Based Stemness Index
OCLR	One-Class Logistic Regression
PBS	Phosphate-Buffered Saline
PCBC	Progenitor Cell Biology Consortium
PI	Propidium Iodide
ROC	Receiver Operating Characteristic
RT-qPCR	real-time quantitative polymerase chain reaction
SI	Staining Index
TFs	Transcription Factors
TMB	Tumor Mutational Burden
TRIM59	Tripartite Motif Containing 59
TSS	Transcription Start Site
TST	Thiostrepton
VST	Variance Stabilizing Transformation
WGCNA	Weighted Gene Co-expression Network Analysis
